# Soil Weathering and Nutrient Dynamics in Response to Land-Use Change Following Forest Conversion to Tea Plantations

**DOI:** 10.3390/plants15050747

**Published:** 2026-02-28

**Authors:** Nan Li, Binbin Shen, Abdelkader Bassiony, Yang Liu, Jianwu Li, Li Ruan

**Affiliations:** 1 State Key Laboratory for Development and Utilization of Forest Food Resources, Zhejiang A&F University, Hangzhou 311300, China; 15700181820@163.com (N.L.); 18368852270@163.com (B.S.); 2 Institute of Sericulture and Tea, Zhejiang Academy of Agricultural Sciences, Hangzhou 310021, China; abdelkader.bassiony@sci.svu.edu.eg (A.B.); liuyang@zaas.ac.cn (Y.L.); 3 Botany and Microbiology Department, Faculty of Science, Qena University, Qena 83523, Egypt

**Keywords:** soil chemical weathering, soil nutrient dynamics, tea plantation conversion, vertical soil profile

## Abstract

Forests’ conversion to tea plantations is a land use transition type with high economic value in China. How this conversion affects soil weathering and nutrient characteristics remains unclear. Here, we selected six soil profiles (three pairs) from representative tea plantations and adjacent forests in China. We quantified the weathering intensity (chemical index of alteration (CIA), base-to-alumina ratio (ba), and weathering index of Parker (WIP)) by soil geography and elemental geochemistry methods and revealed nutrient distributions along with soil profiles. The results showed that soluble elements (such as K_2_O, CaO, MgO and Na_2_O) and SiO_2_ were noticeably leached, while Al_2_O_3_ and P_2_O_5_ were enriched. The geochemical indices showed that the soil profiles of tea plantations (CIA: 80.6%, ba: 0.3 and WIP: 34.6%) experienced stronger chemical weathering than those of forest soils (CIA: 76.0%, ba: 0.4 and WIP: 39.7%). The mean sensitivity indexes (SI) of soil pH, soil organic matter (SOM), total phosphorus (TP) and total potassium (TK) were −7.0%, −24.8%, 53.7% and −8.6%, respectively. This reflected that tea plantations would lead to soil acidification, organic matter depletion, phosphorus enrichment, and potassium deficiency. Our work underscores the significant impact of anthropogenic tea-garden cultivation on pedogenesis; future management must emphasize rational fertilization to prevent soil degradation.

## 1. Introduction

Land-use change alters the soil’s physical and chemical properties, thereby influencing mineral weathering rates and nutrient mobility [1,2]. Shifts in vegetation cover and soil management practices can accelerate the release and leaching of certain elements or promote nutrient accumulation and transformation-processes that critically affect ecosystem productivity and long-term sustainability [3,4]. In China, one of the most ecologically significant land-use transitions is the conversion of natural forests to tea (*Camellia sinensis* (L.) O.Kuntze) plantations, driven largely by the higher economic returns of tea cultivation [5]. Such forest-to-plantation conversion has been reported to undermine soil ecological functions, notably through significant reductions in soil organic carbon stocks in surface soils [6,7,8,9,10,11]. As the world’s second most consumed non-alcoholic beverage after water, tea occupies a pivotal role in the global agricultural economy. Tea is predominantly cultivated in subtropical and tropical regions. According to the Food and Agriculture Organization (FAO) of the United Nations, in 2023, China accounted for 63.6% of the global tea plantation area (3.4 million hectares) and 49.2% of total tea production (3.3 million tons) [12]. Notably, the tea-cultivation area in China has expanded by over 150.0% since the early 21st century [12]. Despite its substantial economic value and role as a major cash crop, tea plantation expansion has raised increasing concerns about soil sustainability, particularly in relation to soil acidification and nutrient imbalance under intensive management [13,14].

Although recent studies have identified general regional trends in soil weathering and nutrient responses to land-use change, most existing work has focused primarily on surface soils, emphasizing changes in soil acid–base balance (pH), soil organic matter (SOM) pools, and the dynamics of major macronutrients such as nitrogen (N), phosphorus (P), and potassium (K). As a result, a systematic understanding of the coupled relationships between chemical weathering and nutrient redistribution across entire soil profiles remains limited [15,16]. Moreover, existing data are drawn primarily from conventional croplands or orchards, while the specific conversion of natural forests to tea plantations, a practice characterized by intense fertilization and strong soil acidification, remains underexplored at the profile scale [14,17]. The conversion of natural forests to tea plantations is typically accompanied by intensified management and prolonged soil acidification, processes that are likely to modify soil weathering intensity and alter patterns of nutrient migration within soil profiles [18,19]. In both natural and agricultural soils, chemical weathering is commonly evaluated using geochemical indices such as the chemical index of alteration (CIA), the weathering index of Parker (WIP), and the base-to-alumina ratio (ba) [20,21,22]. Compared with single-element indicators, these indices are less affected by differences in parent material because they are derived from elemental geochemical behavior and weathering reactions [21]. CIA reflects the relative enrichment of Al during silicate alteration, WIP indicates the overall loss of mobile elements, and ba describes the retention or leaching of base cations relative to alumina [21,22]. Used together, they provide complementary information on mineral transformation, elemental depletion, and base cation dynamics during soil development. The relative migration rate (Δ) further quantifies elemental gain or loss during pedogenesis. By normalizing elemental changes to a stable reference element, it minimizes the influence of variations in soil texture, bulk density, and moisture, allowing comparison across different sites and management conditions [23]. As a dimensionless metric, it provides a practical way to assess elemental redistribution and potential nutrient loss under different weathering intensities [24]. Despite the widespread use of these indicators, the relationship between weathering intensity (CIA, WIP, ba, and relative migration rate) and both total and plant-available nutrient pools remains poorly constrained. Few studies have directly linked quantitative weathering indices with changes in total and available nutrient pools. Consequently, our ability to predict how accelerated weathering affects nutrient availability is still limited [25].

The objective of this study was to quantify changes in soil weathering and nutrient characteristics following the conversion of forest to tea plantations throughout the entire vertical soil profile, to elucidate the regulation of total versus available nutrient pools by weathering gradients, and to establish geochemical foundations for the ecological sustainability and precision nutrient management of tea plantation systems [26,27].

In this study, we employed a pairwise sampling strategy to collect six soil profiles (three paired) from representative tea plantations and adjacent forests with different soil types in China, where tea plants have been widely planted for at least a few decades. These tea fields were all converted from forests, and each tea plantation plot has the same soil type as the adjacent forest plot. Through soil geography and elemental geochemistry methods, we quantified the weathering intensity by using chemical index of alteration (CIA), base-to-alumina ratio (ba), and weathering index of Parker (WIP), and also examined the nutrient distributions along soil profiles. In addition, relative migration rates (Δ) and sensitivity indices (SI) were calculated to quantitatively evaluate nutrient mobility within the soil profiles. The objective of this study was to test two hypotheses: (1) a tea plantation, as an anthropogenic driver, accelerates soil weathering and desilication-allitization; (2) tea plantations’ impact on soil nutrients depends on nutrient mobilities. This study emphasizes the importance of anthropogenic factors in soil pedogenesis following forest conversion and provides a theoretical basis for prioritizing soil protection during forest-to-tea plantation conversion.

## 2. Results

### 2.1. Pedogeochemical Characteristics of Soil Elements

**Figure 1:**
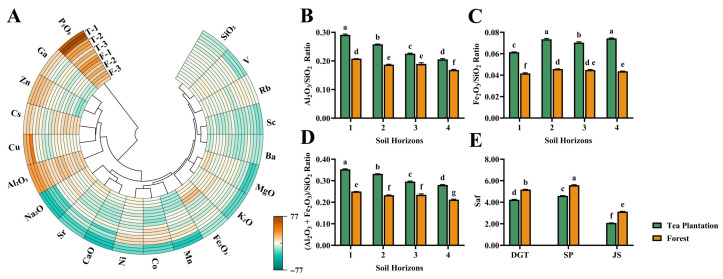
Pedogeochemical properties and weathering indices of tea plantation and adjacent forest soils. (**A**) Relative migration rates (Δ) of major oxides and trace elements along soil profiles, with Ti used as the immobilized reference element and horizon 4 as the reference horizon (Δ = 0). Positive values (shown in orange) indicate enrichment, whereas negative values (shown in teal) indicate depletion relative to the parent material. Profiles T-1 to T-3 represent tea plantations; profiles F-1 to F-3 represent adjacent natural forests. (**B**–**D**) Vertical changes in oxide ratios across soil layers used for tea plantations and forests. (**E**) Changes in the Saf (soil oxide residual coefficient) index along soil profiles for the three tea plantation sites and their paired forest controls. Different lowercase letters indicate significant differences among treatments (*p* < 0.05).

Pedogeochemical signatures revealed distinct land-use dependent patterns in elemental migration. The soluble elements, including K_2_O, CaO, MgO and Na_2_O, showed pronounced leaching in tea plantation surface soils, with an average migration rate of 15.6%, significantly exceeding those in corresponding forest soil horizons. SiO_2_ exhibited a noticeable leaching trend. In tea plantation soils, its migration rates ranged from −10.8% to −1.8%, generally lower than the −4.5% to −0.8% observed in forest soils. In contrast, Al_2_O_3_ and P_2_O_5_ exhibited enrichment trends in surface layers of both land-use types, with P_2_O_5_ migration rates reaching 76.5% in tea plantation A-horizons, substantially higher than the 32.9% observed in forest soils (Figure 1A). Sc, V, Co, Ni, and Ba were preferentially leached from tea plantation top soils, whereas Cu and Zn exhibited accumulation. Fe_2_O_3_ accumulated in the B-horizons of forest soils but showed no similar enrichment in tea plantations. The tea plantation soils underwent silica depletion coupled with iron and aluminum accumulation, evidenced by elevated Al_2_O_3_/SiO_2_ and Fe_2_O_3_/SiO_2_ ratios in surface horizons and higher residual values (Figure 1B–D). The silica–sesquioxide ratio (Saf) values were markedly lower in tea plantation soils (mean = 3.6) than in forest soils (mean = 4.6), representing an average decrease of approximately 21.4% (Figure 1E). Overall, soil geochemistry showed a pattern of silica loss with aluminum and iron enrichment, along with a strong leaching of base cations like potassium, calcium, magnesium, and sodium, and noticeable accumulation of phosphorus in surface layers, highlighting how human management has profoundly altered soil weathering and element migration.

### 2.2. Soil Chemical Weathering Intensity

**Figure 2:**
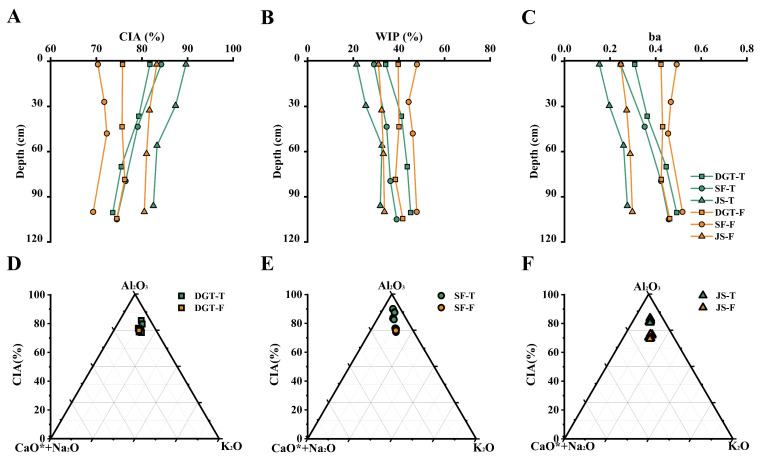
Vertical variations in chemical weathering indices and A-CN-K weathering trajectories in tea plantation and forest soils. Vertical distributions of the Chemical Index of Alteration (CIA, %, **A**), the Weathering Index of Parker (WIP, %, **B**), and the base/alumina ratio (ba, **C**) along soil profiles (0–120 cm) for tea plantation (T) and adjacent forest (F) soils at the DGT, SF, and JS sites. Depth increases downward. Panels (**D**–**F**) show A-CN-K ternary diagrams based on molar proportions of major oxides for paired tea plantation and forest profiles at the DGT, SF, and JS sites, respectively.

Geochemical weathering indices indicate that soils in the study area have undergone moderate to strong chemical weathering. The mean CIA was significantly higher in tea plantation soil (80.6%) than in adjacent forest soil (76.0%) (Figure 2A). This CIA elevation exceeds the typical range for subtropical forest soil (60.0–70.0%). CIA values across all tea plantation profiles ranged from 73.6% to 89.6%. The *ba* ratio varied from 0.2 to 0.5, with the mean value declining from 0.4 in forest soils to 0.3 in tea plantations, reflecting accelerated base cation leaching. Similarly, the average *WIP* decreased from 39.7% in forests to 34.6% in tea plantations, demonstrating more rapid depletion of mobile base elements (Figure 2B,C). The A-CN-K (Al_2_O_3_-CaO* + Na_2_O-K_2_O) ternary diagram is based on the molar proportions of Al_2_O_3_, CaO* + Na_2_O, and K_2_O. The diagram shows that all of the samples fall along the plagioclase-K-feldspar weathering trend. Samples from tea plantations are closer to the Al_2_O_3_ (A) apex, while samples from forests are closer to the A-CN join (Figure 2D–F). This indicates that tea plantation management enhances desilication and the depletion of Ca-Na-bearing phases, driving soil composition toward an Al_2_O_3_-enriched end member.

### 2.3. Soil Nutrient Characteristics

**Figure 3:**
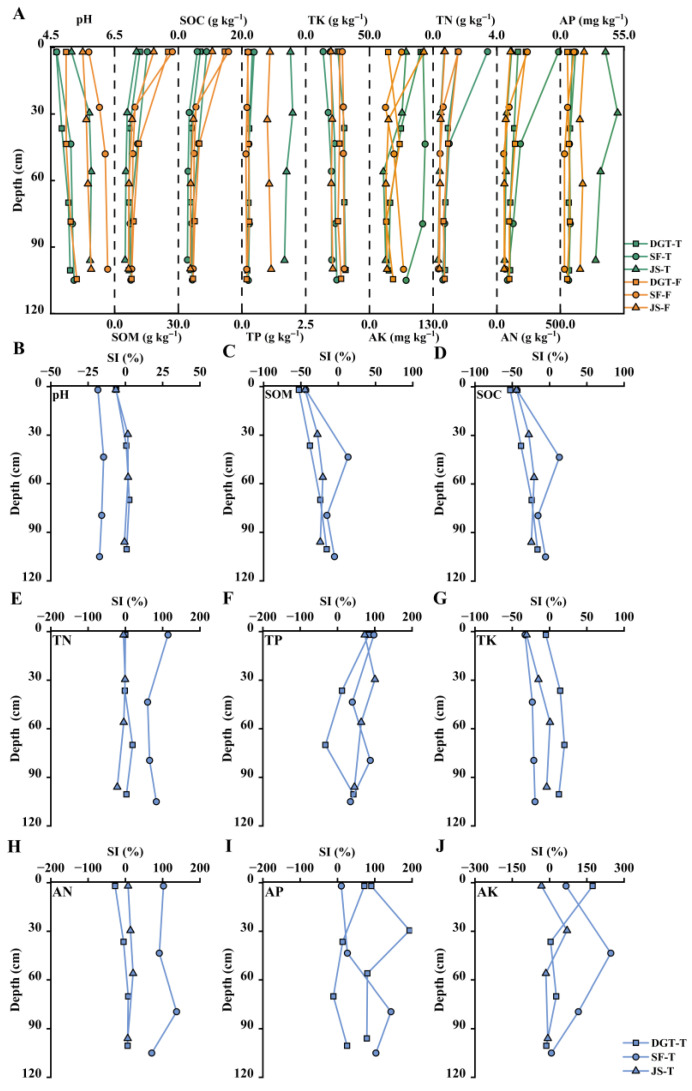
Vertical variations in soil nutrient contents and sensitivity indices following forest conversion to tea plantations. (**A**) Changes in soil physicochemical properties and nutrient contents after conversion from forest to tea plantation. (**B**–**J**) Vertical distributions of the sensitivity index (SI) for pH, soil organic matter (SOM), and total nitrogen (TN), illustrating the relative response of tea plantation soils compared with adjacent forest soils.

Surface soil SOM and SOC were consistently lower in tea plantations than in adjacent forest soils (Figure 3A). In tea plantations, SOM ranged from 10.3 to 15.3 g kg^−1^ and SOC from 6.0 to 8.9 g kg^−1^, whereas forest soils showed higher SOM contents of 18.4 to 27.4 g kg^−1^ and SOC contents of 10.7 to 15.8 g kg^−1^. The greatest differences in surface SOM and SOC between tea plantation and forest soils were observed at the DGT site, reaching 13.3 g kg^−1^ and 6.9 g kg^−1^, respectively. Compared with forest soils, tea plantation soils were characterized by pronounced phosphorus enrichment and an overall decline in potassium contents, whereas nitrogen contents showed no consistent differences between land-use types (Figure 3A). Total phosphorus (TP) in tea plantation soils ranged from 0.2 to 2.0 g kg^−1^, and available phosphorus (AP) from 7.1 to 49.4 mg kg^−1^, both exceeding the corresponding ranges in forest soils (0.2–1.2 g kg^−1^ and 3.5–20.5 mg kg^−1^, respectively). In contrast, total potassium (TK) contents in tea plantation soils were generally lower than those in forest soils, while available potassium (AK) showed variable site-specific responses. Total and available nitrogen (TN and AN) contents remained broadly comparable between tea plantation and forest soils.

Vertically downward from the soil surface, the soil pH Sensitivity Index (SI) first increased and then decreased. The soil pH SI values of the surface soils were all negative, ranging from −17.7% to −6.2%, indicating the surface acidification of tea-garden soils (Figure 3B). The SOM and SOC SI values of the surface soils were consistently negative (SOM: −52.7% to −5.0%; SOC: −5.3% to −52.7%), signifying their severe depletion relative to forest baselines (Figure 3C,D). For total nutrient contents, total phosphorus responded most strongly to tea plantation establishment, with a mean sensitivity index of 53.7% (Figure 3F). In contrast, the total potassium SI was predominantly negative, with a mean value of −8.6% (Figure 3G). However, total nitrogen SI exhibited considerable regional variation, ranging from −21.7%to 114.3% (Figure 3E). Overall, available nutrients were more responsive to land-use change than their total pools, as reflected by higher absolute SI values (mean SI of 59.0% for available nutrients versus 34.6% for total nutrients). This difference was especially clear along the soil profile. Surface and subsurface soils exhibited stronger responses than deeper layers, with mean SI values of 69.3% and 48.7%, respectively (Figure 3H,I). Among the individual nutrients, available phosphorus exhibited the highest sensitivity (mean SI of 70.8%), followed by available potassium (64.9%), whereas alkali–hydrolyzable nitrogen showed a comparatively weaker response, with a mean SI of 41.3% (Figure 3H,I). Taken together, these results suggest that soil responses to tea plantation management differ markedly among nutrient elements and are strongly depth-dependent, with surface and subsurface layers being more sensitive than deeper soils.

### 2.4. The Relationships Between Weathering and Nutrient Characteristics

**Figure 4:**
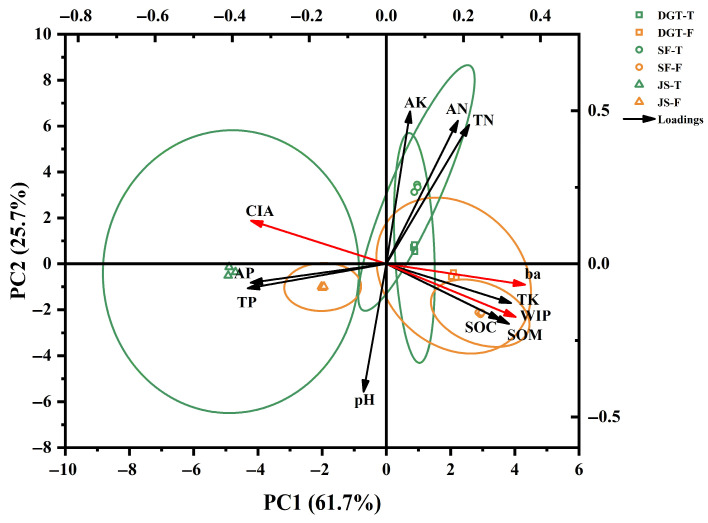
Principal component analysis (PCA) biplot showing how soil chemical weathering indices and nutrient properties are related in the tea plantation and nearby forest soils.

PCA revealed systematic linkages between weathering processes and nutrient cycling in typical tea plantation and forest soil systems (Figure 4). The first two principal components (PC1 and PC2) collectively explained 87.4% of the total variance (PC1: 61.7%; PC2: 25.7%), effectively capturing the dominant patterns of variation within the dataset (Figure 4). For each site, tea plantation and forest soil samples could be clearly separated. The JS site (parent material: Weathered Residual-Colluvium from Basalt) was positioned far from both the DGT site (parent material: Weathered Colluvium from Tuff) and the SF site (parent material: Weathered Colluvium from Tuff).

For the PC1, the projections of total phosphorus (TP) and available phosphorus (AP) were the same as those of CIA, while the projections of total potassium (TK) and available potassium (AK) were opposite to those of CIA. This suggested that positive correlations existed between intense chemical weathering and phosphorus enrichment, whereas weaker weathering environments favored potassium retention. In addition, the projection of soil organic matter (SOM) was opposite to that of CIA along the PC1 axis, indicating that weaker weathering environments favored SOM retention. Sample distribution reinforced these relationships. Soils from the JS tea plantation site clustered in the positive PC1 sector, corresponding to their higher CIA values (82.5–89.6%) and elevated available phosphorus concentrations (30.5–49.4 mg kg^−1^). Conversely, samples from the DGT tea plantation were distributed in the negative PC1 sector, consistent with their lower CIA values (73.6–81.7%) and greater total potassium content (25.3–31.3 g kg^−1^).

For the PC1, the projection of CIA was the same as that of pH, while the projections of *WIP* and *ba* were opposite to that of pH. This suggested that increased soil weathering could intensify acidification, and in turn, acidification could accelerate soil weathering. Regardless of whether along the PC1 or PC2 axis, the projections of available nitrogen (AN) and available potassium (AK) were opposite to that of pH, while the projection of available phosphorus (AP) was the same as that of pH. This reveals that soil acidification increased the availability of potassium and nitrogen in soils, while it reduced the availability of phosphorus. The effect of pH on soil nutrients was consistent with that of weathering; however, it was more pronounced and direct, as it was simultaneously reflected in both PC1 and PC2.

## 3. Discussion

### 3.1. Intensified Soil Acidification and Enhanced Chemical Weathering and Desilication–Fe/Al Enrichment in Tea Plantation Soils

Following the conversion of forests into tea plantations, soils became more acidic, with both chemical weathering and desilication-Fe/Al enrichment clearly intensified [14]. At the same time, the dominant drivers of soil formation shifted from natural factors-primarily climate, organisms, and parent material—to human activities such as fertilization, tillage, and vegetation management.

The repeated and long-term application of fertilizers to maintain high yields in managed tea plantations is a major factor contributing to soil acidification [13]. In addition, tea roots, which tolerate high levels of aluminum, release organic acids that further lower the pH of the rhizosphere and surface soils. Under these persistently acidic conditions, primary silicate minerals break down more quickly, and H^+^ progressively displace alkali and alkaline-earth cations (K^+^, Ca^2+^, Mg^2+^, and Na^+^) from the mineral lattices, promoting the loss of soluble base cations [28,29]. This is evident from the observed declines in ba and WIP values, along with increases in CIA values [22]. As base cations are used up, the soil’s natural buffering ability becomes weaker, which lets acidification keep happening [30]. Silicon that comes from breaking down minerals is easier to leach down, while iron and aluminum oxides (Fe_2_O_3_ and Al_2_O_3_), which are relatively stable, build up [31]. This makes desilication and Fe/Al enrichment even stronger. The weathering and desilication-*Fe/Al* enrichment make the soil’s base cation reserves even smaller, making it less able to neutralize acids that come in. These processes work together to create a feedback loop. Acidification speeds up chemical weathering and desilication-Fe/Al enrichment, which makes soil acidification worse by causing base cation loss to continue [32].

### 3.2. Reorganization of Soil Nutrient Cycling Following Forest-to-Tea Plantation Conversion: Depletion of Organic Matter, Phosphorus Accumulation, and Potassium Depletion

Compared with adjacent forest land, the soil of tea gardens transformed from forest land generally exhibits characteristics of decreased soil organic matter, increased phosphorus enrichment, and relatively depleted potassium. This indicates that land use changes have had a significant impact on the original nutrient cycling [10,33]. During the forest stage, continuous input of litter and relatively slow turnover of organic matter were conducive to the accumulation of nutrients in the surface soil [34,35]; but after transforming into tea gardens, changes in vegetation structure and management methods occurred, with reduced input of litter, and repeated removal of aboveground biomass, which accelerated the consumption process of soil organic matter [17,35]. At the same time, soil acidification caused by long-term fertilization and human activities further exacerbated the nutrient differentiation [36,37]. Under acidic conditions, the weathering of potassium-containing minerals and the activation and leaching of potassium are more active, gradually weakening the potassium pool in the tea garden soil; in contrast, phosphorus is more easily fixed or adsorbed in acidic soils rich in iron and aluminum, thus continuously accumulating in the surface soil [38,39,40]. Overall, the combined effect of biogeochemical processes and human interference led to the reorganization of soil nutrient structure after the transformation from forest land to tea gardens, ultimately forming a nutrient pattern characterized by low organic matter, phosphorus enrichment, and relatively depleted potassium [18,41].

### 3.3. Influence of Parent Material on Weathering and Nutrient Characteristics in Tea Plantation Soils

The inherent geochemical properties of different parent materials are closely related to the weathering and nutrient content of tea garden soils [42,43]. Principal component analysis clearly reveals that soil samples from different parent materials are clearly separated along the gradient dominated by CIA and ba (such as PC1) [15,44]. This directly visualizes that the degree of weathering drives these soil property differences [31]. The soil samples of DGT and SF from the tuff parent material show a high degree of similarity in weathering and nutrient characteristics, located in the positive direction of PC1, while the JS sample from the basalt parent material is significantly separated and located in the positive direction of PC1, with a significantly stronger CIA value [42]. Basalt is rich in iron-magnesium minerals and is more prone to weathering under acidic conditions, and its original phosphorus content is higher, which leads to the extremely strong weathering signal (the highest CIA value) and an abnormally prominent phosphorus enrichment phenomenon in the JS tea garden soil, driven by artificial acidification [42,43]. In contrast, the soil developed from the tuff parent material has a relatively abundant initial base content and has a certain buffering capacity against acidification. Although the weathering degree and phosphorus accumulation of the soil from the adjacent forest land are enhanced, the amplitude is weaker than that of the basalt soil [44,45]. Although tea plantation soils derived from different parent materials consistently show declines in pH, enhanced leaching of base cations, and intensified desilication, accompanied by iron and aluminum enrichment after conversion from forest land, variations in parent material chemistry result in differing weathering intensities and patterns of nutrient accumulation [45,46].

## 4. Materials and Methods

### 4.1. Study Area

**Figure 5:**
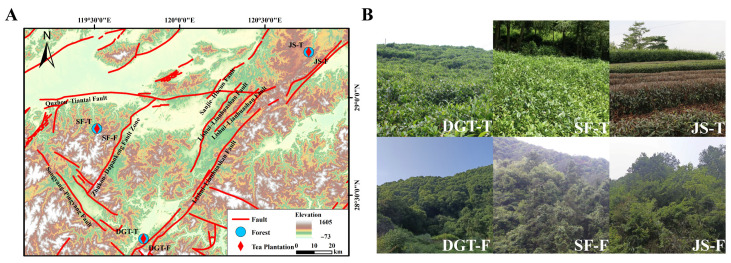
Location of the study area and landscape characteristics of paired tea plantations and adjacent forest sites. (**A**) Geographic location of the sampling sites within the study region. (**B**) Landscape views of tea plantations and their adjacent forest sites at the DGT, SF, and JS locations.

The research was conducted in Zhejiang Province, southeastern China (119°30′30″ E–120°44′29″ E, 28°30′00″ N–29°22′00″ N; Figure 5). It belongs to the southern margin of the subtropical monsoon climate zone (annual mean precipitation: 1689.6 mm, annual mean temperature 18.8 °C, and annual average sunshine duration: 1726.8 h). The lowest monthly mean air temperature is −7.0 °C in January, and the highest value is 42.8 °C in August [47].

The parent materials of the study sites were inferred based on regional geological information and field observations of soil profile characteristics (e.g., exposing fresh rock surfaces with a geological hammer to aid lithological identification) [48]. Soil types were classified according to the USDA Soil Taxonomy [49]. The soils at DGT and SP are derived from tuff. The contents of quartz and feldspar minerals are relatively high, with relatively high SiO_2_ content. In contrast, the parent rock at JS is basalt, a mafic extrusive rock. It is composed mainly of highly weatherable ferromagnesian minerals such as pyroxene, olivine, and basic plagioclase. This material is rich in Ca, Mg, and Fe, but relatively low in silica.

At the DGT site, every year in November, approximately 700.0 kg ha^−1^ of compound fertilizer is applied all at once as base fertilizer (broadcast application). Compost or accumulated fertilizer is typically incorporated around November as part of routine management. Dry tea yields range from 1.6 to 2.0 t ha^−1^ yr^−1^. Deep pruning is carried out after the spring tea harvest each year. Over time, the tea plantation forms a continuous and dense canopy with relatively uniform bush structure, reflecting long-term stable management. The adjacent forest control (DGT-F) is a subtropical evergreen broad-leaved forest with a well-developed understory and a thick litter layer.

At the SF site, every year in November, approximately 900.0 kg ha^−1^ of compound fertilizer is applied all at once as base fertilizer (broadcast application). Compost is also added around November. Dry tea yields range from 1.5 to 1.8 t ha^−1^ yr^−1^. Deep pruning follows the spring harvest each year. This tea plantation showed a more heterogeneous canopy, with scattered trees and noticeable variation in bush growth across the site. The corresponding forest control (SF-F) consists of a mixed stand dominated by the camphor tree, with a relatively complex vertical structure and substantial litter accumulation.

The JS site follows a similar management pattern but received higher fertilizer inputs, approximately 1200.0 kg ha^−1^ yr^−1^. Compost is likewise applied around November. Dry tea yields range from 2.1 to 2.7 t ha^−1^ yr^−1^. Deep pruning is conducted after the spring harvest each year. The tea plantation exhibits a compact canopy arranged in clearly defined rows, reflecting relatively intensive management. The adjacent forest control (JS-F) is located at a relatively higher elevation and shows a mixed forest structure, with well-developed understory vegetation and litter layers.

### 4.2. Soil Sampling and Laboratory Analysis

**Figure 6:**
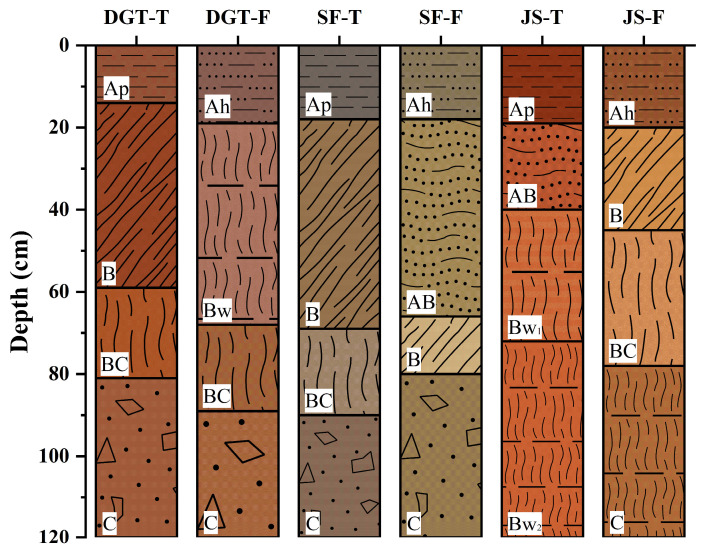
Schematic diagrams of soil profiles of tea plantation and adjacent forest soils. Horizon designations follow standard pedological classification: Ap or Ah represents the humus horizon in the topmost layer; B and Bw represent the illuvial and cambic horizon, respectively, Bw1 and Bw2 denote the upper and lower parts of the cambic horizon, respectively; AB represents the transitional horizon between the surface A horizon (Ap or Ah) and the underlying B horizon and B; BC represents the transitional horizon between B and C; and C represents the parent material horizon.

Three paired sites (DGT, SF, JS) were selected, each comprising one tea plantation and one adjacent forest (Figure 6, Table 1). At each site, 3–5 soil profiles were trial-excavated and screened based on morphological characteristics, landscape position, and parent material consistency. Profile selection was based on integrated field assessment of morphological characteristics, rapid field diagnostics, landscape position, parent material consistency, and land-use history (Figures S1–S3, Table S1). One representative profile per land-use type was designated as the typical profile (Table 2). Soil samples were collected by genetic horizon from the bottom to the top of each profile. Three replicate samples were collected from each horizon and analyzed separately. A total of 72 individual measurements were obtained (Tables S2–S4). These measurements were derived from six soil profiles (three tea plantations and three adjacent forests), each divided into four genetic horizons.

Laboratory procedures included: (1) Soil samples were dried at room temperature and gently crushed using a wooden pestle and mortar, and then sieved through 10-, 60-, and 100-mesh sieves. (2) The pH value, organic matter, and fertility of soils were determined as described by Zhang and Gong [50]. The pH was determined potentiometrically at a 2.5:1 water–to–soil ratio, organic matter was measured by potassium dichromate oxidation, total nitrogen by the Kjeldahl method, available nitrogen by alkaline diffusion, total phosphorus by acid digestion with Mo-Sb colorimetry, available phosphorus by NaHCO_3_ extraction, total potassium by HNO_3_ digestion–ICP–MS, and available potassium by NH_4_OAc extraction. (3) Major element analysis: 100-mesh samples were analyzed using an inductively coupled plasma mass spectrometer (ICP–MS) at the State Key Laboratory of Ore Deposit Geochemistry, Institute of Geochemistry in the Chinese Academy of Science [51]. Standard reference materials were GSR-3, BCR-1, GXR-5 and GXR-6. Analytical uncertainties were less than ±5% for the trace elements.

### 4.3. Data Processing and Analysis

#### 4.3.1. Data Processing

Data processing was performed using Microsoft Excel 2016 (Microsoft Inc., Redmond, WA, USA) for descriptive statistics, including means and standard deviations. Multivariate analysis and visualization were conducted in Origin 2024 (OriginLab Inc., Northampton, MA, USA), with principal component analysis (PCA) applied to identify key patterns. Element migration rates were calculated to quantify pedogenic mobility, while chemical weathering intensity was assessed using standard geochemical indices: Chemical Index of Alteration (CIA), base/alumina ratio (ba), and Weathering Index of Alteration (WIP). The Sensitivity Index (SI) was employed to evaluate nutrient responsiveness to land-use conversion from natural forest to tea plantation.

Stable reference elements (e.g., Ti) were used to evaluate the relative enrichment or depletion of other elements. Xs and Is denote the concentrations of element X and the reference element in the soil sample, respectively, whereas Xp and Ip refer to their concentrations in the parent material [52]. The element migration rate (Δ) was calculated following the immobile element mass balance approach. Δ values are expressed as percentages (%) and reflect the relative gain or loss of element X compared with the parent material [52]. Negative Δ values indicate depletion relative to the reference element, while positive values indicate enrichment. In this study, Ti was selected as the reference element, and Δ was calculated as follows:

(1) $$\Delta \;=\;(\frac{\frac{\mathrm{Xs}}{\mathrm{Is}}}{\frac{Xp}{\mathrm{Ip}}}-1)\;\times \;100\%$$

The silica–sesquioxide ratio (Saf) was used as an indicator of soil weathering intensity. Under comparable parent material conditions, lower Saf values generally reflect stronger chemical weathering, associated with enhanced silica leaching and the relative enrichment of iron and aluminum during soil development [53]. The Saf was calculated as

(2) $$Saf=\frac{{\mathrm{SiO}}_{2}}{{\mathrm{Al}}_{2}O_{3}\;{+\mathrm{Fe}}_{2}O_{3}}$$

The following thresholds are established by conventional interpretation: the higher CIA value indicates stronger weathering [54]. The CIA was calculated as

(3) $$\mathrm{CIA}=\frac{{\mathrm{Al}}_{2}O_{3}}{{\mathrm{Al}}_{2}O_{3}+{\mathrm{CaO}}^{*}+{\mathrm{Na}}_{2}O+K_{2}O}\;\times \;100\%$$

The base/alumina ratio (ba) is defined as the molar ratio of base cation oxides Na_2_O, K_2_O, CaO, and MgO to aluminum oxide Al_2_O_3_ in soils [55]. The ba value is calculated using the following formula:

(4) $$\mathrm{ba}\;=\;\frac{{\mathrm{Na}}_{2}O\;+\;K_{2}O\;+\;\mathrm{CaO}\;+\;\mathrm{MgO}}{{\mathrm{Al}}_{2}O_{3}}$$

The WIP was calculated to assess the weathering stage [56]. Lower WIP values indicate greater leaching of mobile base elements and, consequently, more intense chemical weathering. CaO* means CaO in silicates, and since all the samples in this study have n(CaO) < n(Na_2_O), n(CaO) is used as the sample CaO^*^ in the calculation [57]. The index is calculated as follows:

(5) $$\mathrm{WIP}\;=\;(\frac{{2\mathrm{Na}}_{2}O}{0.35}+\frac{\mathrm{MgO}}{0.90}+\frac{{2K}_{2}O}{0.25}+\frac{{\mathrm{CaO}}^{*}}{0.70})\;\times \;100\%$$

The Sensitivity Index (SI) was employed to evaluate the differences in the response magnitude of various nutrient components across soil profile layers to land-use changes [58,59]. In this assessment, Q_1_ represents the nutrient content in a specific soil layer of natural forest, while Q_2_ denotes the corresponding nutrient content in the same layer under other land-use types. The *SI* was calculated as follows:

(6) $$\mathrm{SI}\;=\;\frac{{(Q}_{2}-Q_{1})}{Q_{1}}\;\times \;100\%$$

#### 4.3.2. Multivariate Analysis

All statistical analyses were performed using SPSS software (version 21.0; IBM, Armonk, NY, USA) and GraphPad Prism 10 (for line charts). Principal component analysis (PCA) was performed using OriginPro 2024 (OriginLab Corporation, Northampton, MA, USA) with the correlation matrix. Variables were standardized to account for differences in measurement units. The first two principal components were used to construct a biplot showing sample scores and variable loadings. All measurements were performed with three replicates. Differences between treatments were assessed using one-way analysis of variance followed by Duncan’s test, with significance set at *p* < 0.05.

## 5. Conclusions

Forest conversion to tea plantations is very common in China. Our study offered geochemical evidence of changes in soil weathering and nutrient characteristics following this conversion. We found that tea plantations strongly accelerated chemical weathering and altered nutrient cycling throughout the soil profile, which was manifested mainly as follows: (1) tea plantations accelerated soil weathering (*CIA* was increased by 6.0%, *WIP* and *ba* were decreased by 13.0% and 16.4%) and intensified desilicification and allitization (*Saf* was decreased by 21.4%); (2) tea plantations accelerated soil acidification (pH was decreased 0.4), depleted organic matter (SOM was decreased by 31.9%) and mobile elements like potassium (total K was decreased by 8.9%), and enriched immobile elements like phosphorus (total P was increased by 69.1%). This study highlights the decisive role of anthropogenic activities on soil pedogenesis. Furthermore, parent rock characteristics also influence the intensity of weathering. Soils derived from tuff are mainly composed of relatively stable silicate minerals with high quartz content, exhibiting some resistance to weathering. In contrast, basalt typically shows stronger weathering reactions. Its higher content of readily weatherable ferromagnesian minerals enhances chemical alteration, and its interconnected vesicular structure further facilitates weathering. The unreasonable fertilization management, harvesting practices, and recurrent human disturbances associated with tea plantations are likely the key drivers of the marked divergence during the conversion of forest to tea plantations. Future management strategies should aim to mitigate acidification, reduce nutrient leaching, and promote balanced nutrient supply to ensure long term soil health and ecosystem resilience.

## Figures and Tables

**Table 1:** General characteristics of soil profiles from tea plantations and adjacent forests in China.

Designation	Location	Soil Classification (USDA Soil Taxonomy, 2022)	Topographic Position	Dominant Species	Erosion Status	Parent Material
DGT-T	Dagangtou Town, Liandu District, Lishui City, Zhejiang Province	Hapludults	Hills, gentle slope, mid-slope position	*Camellia sinensis* (L.) Kuntze.	Slight sheet erosion	Weathered colluvium from tuff
DGT-F	Dagangtou Town, Liandu District, Lishui City, Zhejiang Province	Hapludults	Hills, gentle slope, mid-slope position	*Machilus thunbergii Siebold & Zucc.*	No obvious erosion	Weathered colluvium from tuff
SF-T	Shafan Township, Wucheng District, Jinhua City, Zhejiang Province	Paleudalfs	Mountains, gentle slope, mid-slope	*Camellia sinensis* (L.) Kuntze.	Slight rill erosion	Weathered colluvium from tuff
SF-F	Shafan Township, Wucheng District, Jinhua City, Zhejiang Province	Paleudalfs	Mountains, gentle slope, mid-slope	*Cinnamomum camphora* (L.) J. Presl	Very slight erosion	Weathered colluvium from tuff
JS-T	Jianshan Town, Pan’an County, Jinhua City, Zhejiang Province	Plinthudults	High hills, gentle slope, lower slope	*Camellia sinensis* (L.) Kuntze.	Moderate sheet erosion, locally with rill erosion	Weathered residual–colluvium from basalt
JS-F	Jianshan Town, Pan’an County, Jinhua City, Zhejiang Province	Plinthudults	High hills, gentle slope, lower slope	*Ailanthus altissima* (Mill.) Swingle	No obvious erosion	Weathered residual–colluvium from basalt

**Table 2:** Morphological properties and horizon descriptions of soil profiles from tea plantations and adjacent forests in China.

Designation	Genetic Horizon	Color (Moist, Munsell Notation)	Soil Texture	Soil Structure
DGT-T	Ap	5YR 3/3	Loam	Granular
	B	5YR 4/4	Clay loam	Angular blocky
	BC	5YR 4/3	Clay loam	Angular blocky
	C	5YR4/4	Gravelly clay	Subangular blocky
DGT-F	Ah	5YR 4/3	Loam	Granular
	Bw	5YR 5/4	Clay loam	Crumb
	BC	5YR 5/6	Clay loam	Angular blocky
	C	10YR 6/4	Clay loam	Granular
SF-T	Ap	10YR 3/1	Silt loam	Granular
	B	10YR 4/6	Loam	Crumb
	BC	10YR 5/6	Clay loam	Angular blocky
	C	10YR 3/6	Clay loam	Angular blocky
SF-F	Ah	2.5Y 4/3	Loam	Granular
	AB	2.5Y 6/4	Clay loam	Angular blocky
	B	2.5Y 6/3	Clay loam	Angular blocky
	C	2.5Y 7/6	Sandy clay loam	Granular
JS-T	Ap	5YR 3/3	Clay loam	Fine granular
	AB	5YR 4/4	Clay loam	Angular blocky
	Bw_1_	5YR 4/3	Clay	Angular blocky
	Bw_2_	7.5YR 4/4	Clay	Subangular blocky
JS-F	Ah	5YR 4/4	Clay loam	Granular
	B	5YR 5/4	Clay loam	Crumb
	BC	5YR 5/3	Clay loam	Angular blocky
	C	5YR 4/3	Clay	Angular blocky

## Data Availability

All data are included in this paper. Additional information can be provided upon request to the corresponding author.

## Supplementary Materials

The following supporting information can be downloaded at: https://www.mdpi.com/article/10.3390/plants15050747/s1, Figure S1. Schematic diagram of the field survey at the DGT site using soil geography methods; Figure S2. Schematic diagram of the field survey at the SF site using soil geography methods; Figure S3. Schematic diagram of the field survey at the JS site using soil geography methods; Table S1. Soil Profiles Field Description; Table S2. Nutrient contents of the soil profiles; Table S3. Major element concentrations in soil profiles; Table S4. Trace element concentrations in soil profiles.

## Abbreviations

The following abbreviations are used in this manuscript:

*CIA*	Chemical Index of Alteration
*WIP*	Weathering Index of Parker
*ba*	Base-to-alumina ratio
*Saf*	Silica–sesquioxide ratio
SOM	Soil Organic Matter
TN	Total Nitrogen
AN	Available Nitrogen
TP	Total Phosphorus
AP	Available Phosphorus
TK	Total Potassium
AK	Available Potassium
*SI*	Sensitivity Index
PCA	Principal Component Analysis
ICP-MS	Inductively Coupled Plasma-Mass Spectrometry
ICP-AES	Inductively Coupled Plasma-Atomic Emission
