# Supplementary material for: Soil Weathering and Nutrient Dynamics in Response to Land-Use Change Following Forest Conversion to Tea Plantations
Source: Plants (Basel). 2026 Feb 28;15(5):747. doi: 10.3390/plants15050747 (PMC12987252; doi:10.3390/plants15050747)
Supplement: Supplementary file 1 [file plants-15-00747-s001.zip › plants-4128233-supplementary.pdf]

Supplementary Material:

Soil Weathering and Nutrient Dynamics in Response to Land-Use Change Following Forest Conversion to Tea Plantations

Nan Li[1,†], Binbin Shen[1,†], Abdelkader Bassiony[2,3], Yang Liu[2], Jianwu Li[1,*], Li Ruan[2,*]

1 Zhejiang A&F University, Hangzhou 311300, China
2 Institute of Sericulture and Tea, Zhejiang Academy of Agricultural Sciences, Hangzhou 310021, China
3 Botany and Microbiology Department, Faculty of Science, South Valley University, Qena, 83523, Egypt
† These authors contributed equally to this work.
* Correspondence: Jianwu Li, jameslee@zafu.edu.cn
Li Ruan, ruanl@zaas.ac.cn

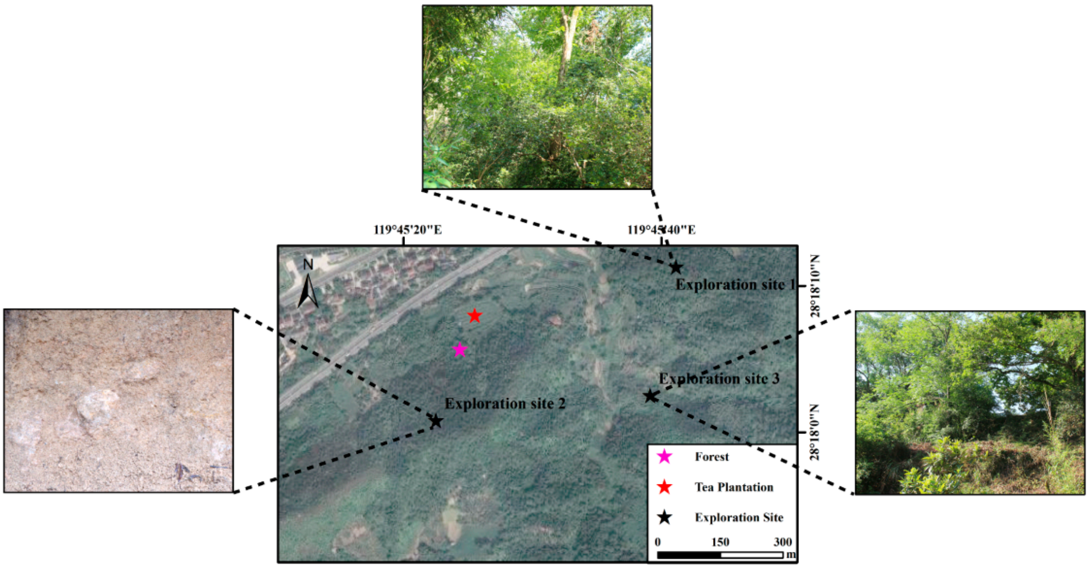

Figure S1. Schematic diagram of the field survey at the DGT site using soil geography methods. Exploration site 1 was excluded because no tea plantations were present nearby. Exploration site 2 was excluded because the parent material is granite rather than tuff. Exploration site 3 was excluded due to different geomorphic position.

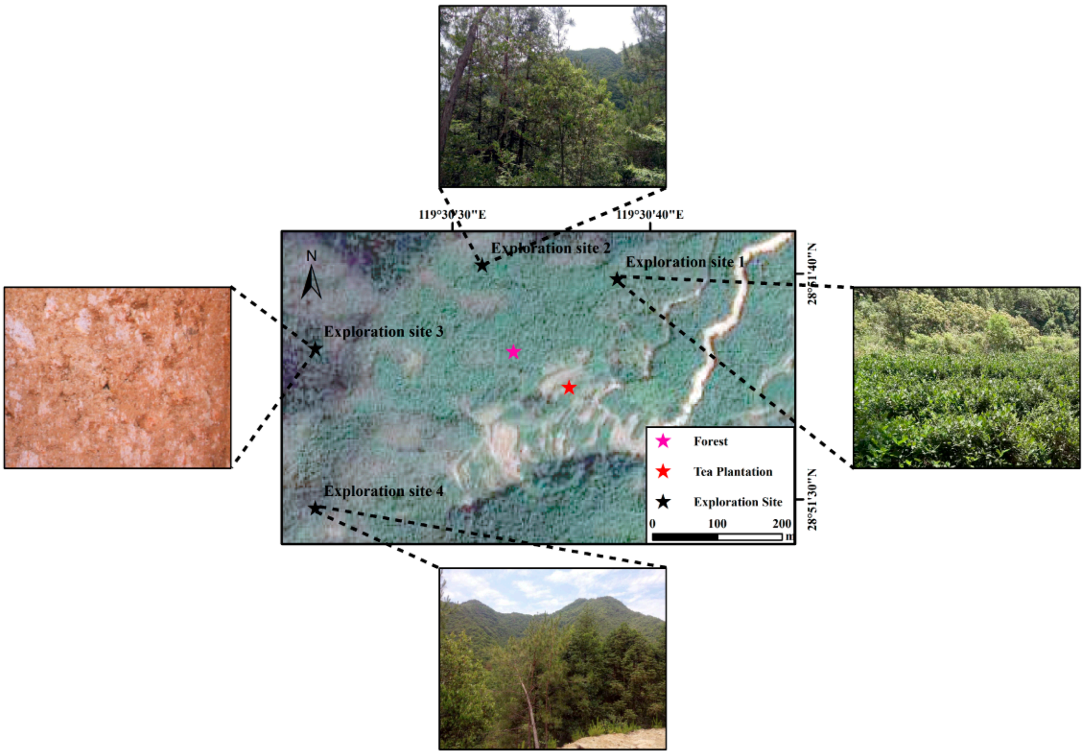

Figure S2. Schematic diagram of the field survey at the SF site using soil geography methods. Exploration site 1 was excluded due to different slope aspect. Exploration site 2 was excluded due to different geomorphic position. Exploration site 3 was excluded because the parent material is granite rather than tuff. Exploration site 4 was excluded due to different geomorphic position.

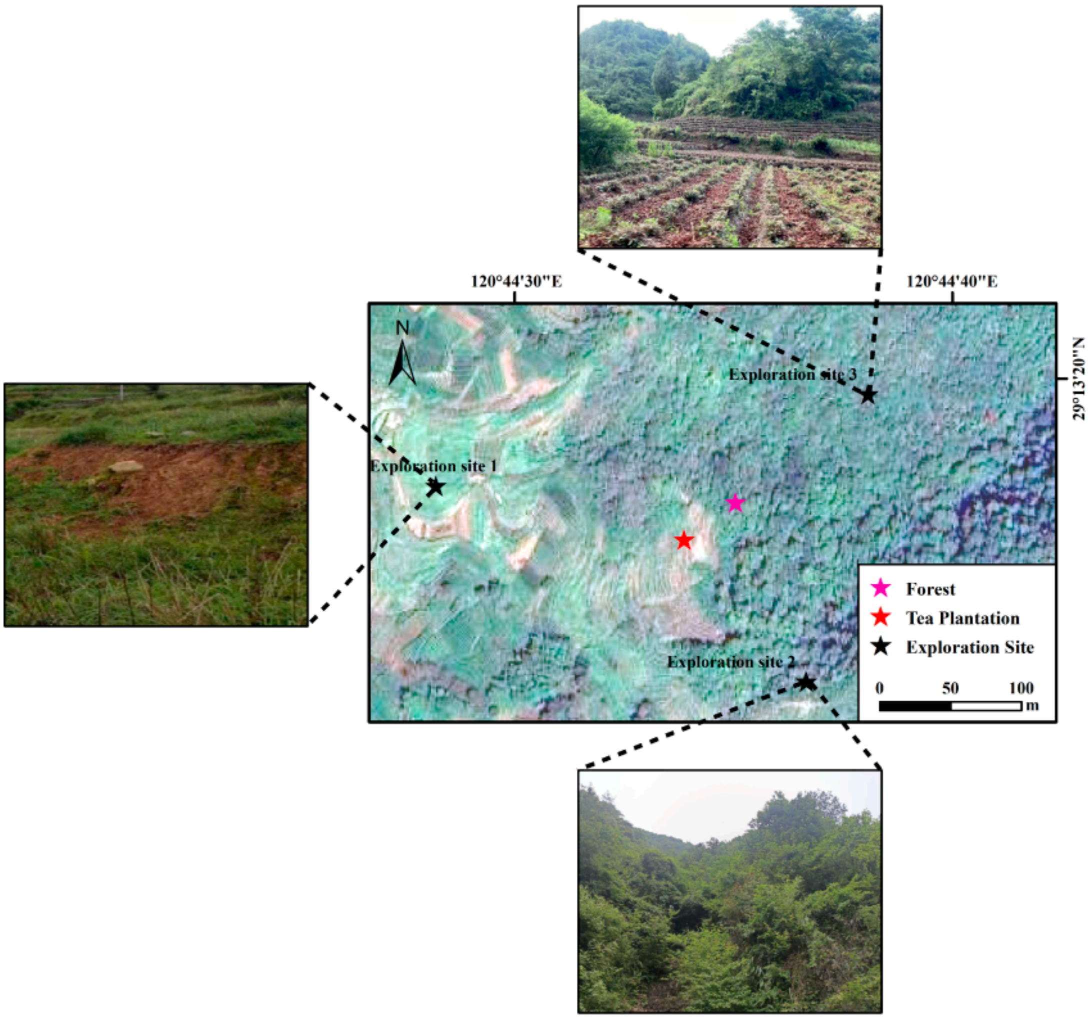

Figure S3. Schematic diagram of the field survey at the JS site using soil geography methods. Exploration site 1 was excluded due to different land use (dryland). Exploration site 2 was excluded due to different geomorphic position. Exploration site 3 was excluded due to different slope aspect.

Table S1. Soil Profiles Field Description

Soil Profile	Field Description
DGT - T	Located on a low mountain hillside in Dagangtou Town, Liandu District, Lishui City, Zhejiang Province, with slight sheet erosion and high surface coverage. The tea plantation has been established for many years. The soil is deep, fine-textured, slightly clayey, with low porosity and poor aeration.
DGT - F	Located on a low mountain hillside in Dagangtou Town, Liandu District, Lishui City, Zhejiang Province, with no noticeable erosion. It is a typical subtropical evergreen broadleaf forest with a thick litter layer and high organic matter content. The soil is undisturbed and shows low weathering. The profile structure is Ah-Bw1-BC-C, and the soil is thick, slightly clayey, with noticeable organic matter accumulation compared to the tea garden.
SF - T	Located on a low mountain gentle slope in Shafan Township, Wucheng District, Jinhua City. The surface shows slight gully erosion, and the tea garden has a long management history with stable fertilization practices. The landscape is uniform. The soil is deep, with a dark surface layer and a yellow-brown color that favors organic matter accumulation. The soil is rich in humus.
SF - F	Located in the same topographical position as SF-T, with minimal erosion. The forest is a mixed forest dominated by camphor, with a complex vegetation structure and noticeable organic matter accumulation in the soil. The soil is dark-colored, with abundant roots, and significant humus accumulation in the surface layer. The lower layer contains much semi-weathered parent material.
JS - T	Located at the base of a high hill in Jianshan Town, Pan'an County, Jinhua City, with moderate sheet erosion and minor gully erosion. The tea plantation is heavily fertilized, and the tea trees are vigorous, with a well-maintained landscape. The soil is deep, fine-textured, slightly clayey, with low porosity and poor aeration.
JS - F	Located at the base of a high hill in Jianshan Town, Pan'an County, Jinhua City, with no noticeable erosion. The forest is a secondary mixed forest with good vegetation cover and minimal human disturbance. The soil is clayey, dark-colored, and has a dense root system. The surface layer is granular, loose, and porous, with dense roots, while the lower layer is more compact.

Table S2. Nutrient contents of the soil profiles.

Designation	soil horizon	Sample Number	pH	SOM（g kg^{-1}）	TN（g kg^{-1}）	AN（g kg^{-1}）	TP（g kg^{-1}）	AP（g kg^{-1}）	TK（g kg^{-1}）	AK（mg kg^{-1}）
DGT - T	DGT-T-1	1	4.690	11.958	1.558	0.189	0.386	0.386	25.106	101.908
		2	4.710	11.936	1.594	0.191	0.427	0.427	25.223	102.606
		3	4.620	11.818	1.563	0.120	0.421	0.421	25.530	109.469
	DGT-T-2	4	4.820	6.334	0.928	0.138	0.277	0.277	29.941	62.118
		5	4.850	7.573	0.948	0.142	0.286	0.286	30.451	61.875
		6	4.860	7.112	0.938	0.130	0.293	0.293	30.388	69.405
	DGT-T-3	7	5.020	6.708	0.769	0.114	0.245	0.245	30.059	37.861
		8	5.040	6.572	0.793	0.105	0.271	0.271	30.270	52.156
		9	5.100	7.028	0.794	0.100	0.251	0.251	30.642	37.233
	DGT-T-4	10	5.120	6.475	0.758	0.109	0.262	0.262	31.478	37.145
		11	5.090	6.616	0.744	0.108	0.250	0.250	30.759	42.502
		12	5.110	6.936	0.755	0.098	0.266	0.266	31.582	38.857
DGT - F	DGT-F-1	13	4.980	24.446	1.571	0.237	0.210	0.210	26.602	37.572
		14	4.970	25.612	1.566	0.230	0.235	0.235	26.586	38.348
		15	4.990	25.469	1.579	0.220	0.226	0.226	26.638	38.858
	DGT-F-2	16	4.980	12.004	0.956	0.145	0.249	0.249	27.025	63.157
		17	4.980	11.472	0.956	0.147	0.283	0.283	26.119	62.251
		18	4.990	10.530	0.943	0.139	0.236	0.236	26.287	60.288
	DGT-F-3	19	5.120	8.998	0.659	0.101	0.370	0.370	25.126	31.630
		20	5.100	8.606	0.647	0.099	0.377	0.377	25.677	30.743
		21	5.150	9.109	0.667	0.096	0.387	0.387	25.063	40.814
	DGT-F-4	22	5.300	8.436	0.751	0.098	0.186	0.186	27.890	38.815
		23	5.350	8.323	0.728	0.097	0.177	0.177	27.769	68.754
		24	5.300	7.101	0.715	0.103	0.182	0.182	27.706	37.528
SF - T	SF-T-1	25	4.680	15.120	3.475	0.494	0.462	0.462	19.051	113.573

		26	4.680	15.258	3.395	0.475	0.492	0.492	19.267	107.518
		27	4.700	15.622	3.409	0.488	0.480	0.480	19.487	107.654
		28	5.130	10.861	1.038	0.192	0.268	0.268	22.292	117.348
	SF-T-2	29	5.110	10.686	1.016	0.192	0.259	0.259	22.618	112.233
		30	5.150	10.867	1.011	0.174	0.296	0.296	23.206	111.716
		31	5.180	7.902	0.737	0.128	0.284	0.284	23.370	108.899
	SF-T-3	32	5.170	7.083	0.730	0.134	0.307	0.307	23.660	108.257
		33	5.190	6.760	0.731	0.126	0.291	0.291	22.812	109.197
		34	5.210	7.607	0.623	0.100	0.256	0.256	24.506	74.487
	SF-T-4	35	5.230	8.034	0.620	0.112	0.238	0.238	23.866	75.893
		36	5.250	7.180	0.624	0.041	0.262	0.262	24.753	74.571
		37	5.630	26.667	1.613	0.240	0.331	0.331	28.490	66.483
	SF-F-1	38	5.700	27.481	1.585	0.234	0.332	0.332	28.558	65.409
		39	5.760	27.363	1.599	0.247	0.325	0.325	28.850	65.516
		40	6.050	9.344	0.647	0.097	0.195	0.195	29.845	31.345
	SF-F-2	41	6.010	9.344	0.644	0.101	0.191	0.191	29.605	33.054
SF - F		42	6.030	9.960	0.628	0.094	0.201	0.201	29.152	34.334
		43	6.210	8.678	0.429	0.050	0.109	0.109	29.522	49.186
	SF-F-3	44	6.250	9.387	0.453	0.057	0.116	0.116	29.602	50.601
		45	6.170	7.646	0.451	0.057	0.113	0.113	29.348	51.162
		46	6.300	8.062	0.344	0.050	0.145	0.145	30.019	70.943
	SF-F-4	47	6.210	7.586	0.336	0.041	0.143	0.143	30.480	69.049
		48	6.350	8.457	0.340	0.105	0.142	0.142	30.318	69.153
		49	5.210	9.004	0.702	0.124	1.655	1.655	13.566	83.237
	JS-T-1	50	5.110	11.147	0.707	0.118	1.606	1.606	13.737	83.732
		51	5.130	10.628	0.707	0.108	1.669	1.669	13.540	57.728
JS - T		52	5.740	5.302	0.476	0.081	2.027	2.027	17.799	81.672
	JS-T-2	53	5.710	6.368	0.477	0.080	1.963	1.963	17.439	57.495
		54	5.680	6.064	0.483	0.080	1.993	1.993	17.733	60.664
	JS-T-3	55	5.750	5.309	0.407	0.077	2.066	2.066	20.109	27.257

		56	5.770	5.123	0.438	0.063	2.008	2.008	20.024	30.667
		57	5.790	5.469	0.420	0.077	1.965	1.965	20.182	28.241
		58	5.690	4.663	0.307	0.056	2.321	2.321	20.719	32.297
	JS-T-4	59	5.760	5.291	0.329	0.066	2.362	2.362	20.397	32.279
		60	5.750	4.836	0.319	0.063	2.254	2.254	20.101	35.769
		61	5.460	17.845	0.742	0.111	1.075	1.075	20.031	108.700
	JS-F-1	62	5.550	18.766	0.744	0.109	1.118	1.118	19.685	111.411
		63	5.500	18.572	0.743	0.106	1.110	1.110	19.282	114.276
		64	5.620	8.322	0.486	0.078	1.030	1.030	20.972	39.455
	JS-F-2	65	5.640	8.155	0.478	0.067	0.968	0.968	20.534	40.183
		66	5.590	8.102	0.482	0.068	0.987	0.987	20.716	37.670
JS - F		67	5.680	6.591	0.439	0.060	1.117	1.117	20.366	33.876
	JS-F-3	68	5.670	6.430	0.443	0.061	1.067	1.067	20.103	33.879
		69	5.650	7.021	0.441	0.059	1.031	1.031	19.522	33.167
		70	5.770	6.366	0.406	0.058	1.196	1.196	21.220	34.580
	JS-F-4	71	5.740	6.197	0.404	0.052	1.152	1.152	21.232	36.124
		72	5.790	6.992	0.410	0.066	1.184	1.184	21.204	37.298

Table S3. Major element concentrations in soil profiles

Designation	soil horizon	Sample Number	Si（g kg-1）	Al（g kg-1）	Fe（g kg-1）	Mg（g kg-1）	Ca（g kg-1）	Na（g kg-1）	Ti（g kg-1）
DGT - T	DGT-T-1	1	242.051	106.794	25.744	4.266	1.244	4.712	3.727
		2	241.450	106.603	25.928	4.231	1.203	4.923	3.699
		3	240.829	107.352	25.564	4.208	1.239	4.852	3.678
	DGT-T-2	4	245.585	109.544	26.565	5.226	1.285	6.008	3.760
		5	243.203	109.308	25.295	5.194	1.295	5.791	3.717
		6	248.084	109.131	25.992	5.197	1.272	5.925	3.657
	DGT-T-3	7	264.459	96.355	25.620	5.441	1.993	7.737	3.767
		8	266.012	97.274	24.439	5.476	1.987	7.464	3.889
		9	264.309	95.332	25.112	5.429	2.009	7.682	3.757
	DGT-T-4	10	269.961	90.507	26.034	5.650	2.110	8.127	3.858
		11	268.169	90.448	26.772	5.616	1.987	7.944	3.870
		12	274.708	88.693	26.278	5.630	2.048	7.748	3.897
DGT - F	DGT-F-1	13	265.614	91.141	22.177	4.397	2.160	7.635	3.592
		14	266.421	91.930	21.931	4.387	2.258	7.938	3.566
		15	265.008	90.170	22.538	4.490	2.200	7.810	3.594
	DGT-F-2	16	274.170	91.197	23.503	4.624	2.191	8.285	3.893
		17	271.234	91.030	23.284	4.613	2.220	7.927	3.893
		18	280.141	92.206	23.222	4.774	2.285	8.014	3.940
	DGT-F-3	19	280.722	92.348	21.371	4.809	2.271	7.924	3.879
		20	282.098	92.003	21.086	4.788	2.352	7.496	3.745
		21	279.289	88.984	20.717	4.832	2.343	7.719	3.663
	DGT-F-4	22	317.774	87.021	23.437	5.052	2.534	8.338	4.069
		23	316.981	91.116	23.102	4.950	2.600	7.868	3.957
		24	316.570	91.485	23.718	5.075	2.586	8.102	3.935
SF - T	SF-T-1	25	249.215	117.465	24.547	3.176	2.036	5.827	4.747

		26	261.788	117.783	25.680	3.280	2.084	5.907	4.889
		27	255.320	118.289	24.905	3.305	1.934	5.682	5.063
		28	273.089	99.361	26.863	4.110	2.578	6.323	4.816
	SF-T-2	29	274.456	99.596	26.825	4.002	2.684	7.533	4.531
		30	277.352	99.667	27.845	4.042	2.710	7.015	5.051
		31	276.317	90.135	26.706	4.655	2.944	7.655	4.969
	SF-T-3	32	276.028	88.857	25.477	4.939	2.927	7.236	4.605
		33	276.275	87.808	24.957	4.817	2.832	7.457	5.104
		34	279.956	87.184	26.915	4.819	3.216	8.394	4.740
	SF-T-4	35	278.237	88.350	26.846	4.747	3.175	9.081	4.564
		36	280.538	87.132	27.447	4.636	3.273	8.583	4.669
		37	292.683	88.421	19.660	2.930	2.894	12.568	7.232
	SF-F-1	38	294.736	88.761	19.538	2.999	2.857	13.218	7.311
		39	297.888	88.427	19.192	2.868	2.945	13.567	7.140
		40	306.245	86.508	39.146	3.044	3.384	9.382	7.606
	SF-F-2	41	311.254	88.872	39.884	2.923	3.351	9.112	7.588
		42	310.553	85.750	39.762	2.957	3.307	9.356	7.556
SF - F		43	310.186	95.454	30.310	3.135	3.823	10.564	7.610
	SF-F-3	44	314.631	94.802	29.692	3.285	3.780	10.641	7.596
		45	313.023	93.893	30.487	3.125	3.692	10.544	7.412
		46	323.108	84.643	28.430	3.035	3.707	11.407	8.069
	SF-F-4	47	319.180	84.420	27.585	2.924	3.627	10.939	8.037
		48	324.650	84.707	28.249	3.024	3.719	11.908	8.067
		49	193.691	139.057	102.779	2.436	1.140	5.044	39.944
	JS-T-1	50	194.313	139.465	102.308	2.403	1.139	4.759	38.881
		51	195.141	142.571	102.216	2.466	1.093	4.899	40.025
JS - T		52	206.039	128.210	137.320	3.152	1.345	4.453	40.795
	JS-T-2	53	207.798	131.342	138.359	3.093	1.493	4.351	39.310
		54	203.944	129.769	138.858	3.175	1.523	4.476	39.780
	JS-T-3	55	218.223	125.173	143.047	3.291	1.675	7.910	40.932

		56	216.503	121.727	140.432	3.411	1.694	8.150	40.909
		57	218.114	122.213	141.345	3.396	1.643	7.780	40.760
		58	229.021	112.885	158.593	3.390	1.697	7.171	41.690
	JS-T-4	59	229.616	114.706	156.688	3.345	1.582	7.276	42.272
		60	226.029	109.913	157.675	3.312	1.667	7.014	42.076
		61	229.381	116.083	80.776	2.492	1.511	7.270	22.247
	JS-F-1	62	229.793	116.507	79.417	2.551	1.517	7.506	22.200
		63	230.802	118.359	78.518	2.451	1.455	7.670	22.217
		64	261.112	111.373	85.749	2.501	1.697	7.543	22.532
	JS-F-2	65	263.295	109.833	87.074	2.442	1.714	7.717	22.078
		66	258.935	108.188	86.772	2.475	1.658	7.738	22.334
JS - F		67	255.851	112.183	93.827	2.764	1.705	8.626	23.470
	JS-F-3	68	254.497	106.513	92.516	2.739	1.722	8.961	24.161
		69	259.821	107.720	92.773	2.725	1.734	8.790	23.503
		70	277.036	107.460	98.590	2.787	2.109	8.011	24.342
	JS-F-4	71	275.635	108.595	100.062	2.800	2.028	7.883	24.586
		72	283.110	105.514	100.293	2.791	2.178	8.104	25.047

Table S4. Trace element concentrations in soil profiles

Designation	soil horizon	Sample Number	Mn (mg kg^{-1})	Sc (mg kg^{-1})	V (mg kg^{-1})	Co (mg kg^{-1})	Ni (mg kg^{-1})	Cu (mg kg^{-1})	Zn (mg kg^{-1})	Ga (mg kg^{-1})	Rb (mg kg^{-1})	Sr (mg kg^{-1})	Cs (mg kg^{-1})	Ba (mg kg^{-1})
DGT - T	DGT-T-1	1	223.401	9.952	70.165	11.246	23.114	21.469	58.466	20.084	99.931	10.076	8.741	198.941
		2	219.346	9.650	72.611	11.341	22.019	21.861	56.660	21.507	99.464	10.823	8.787	193.842
		3	222.378	9.873	69.656	11.291	22.701	21.679	57.750	22.106	99.897	10.004	8.794	197.111
	DGT-T-2	4	356.828	9.941	68.923	12.692	23.910	18.109	53.832	17.391	102.337	11.022	8.878	206.637
		5	362.555	10.479	70.262	12.302	22.010	16.863	52.083	17.881	92.865	12.961	8.557	208.121
		6	360.869	9.770	69.411	12.899	24.816	17.098	53.180	17.665	109.203	12.175	8.690	207.715
	DGT-T-3	7	302.983	10.149	71.863	13.328	23.653	17.762	51.453	19.520	101.756	14.599	9.389	217.860
		8	295.843	9.975	71.268	12.928	25.366	17.139	49.573	19.409	100.047	14.921	9.711	213.617
		9	298.757	10.468	71.026	13.304	24.960	16.576	50.203	18.906	106.160	14.320	9.337	215.580
	DGT-T-4	10	314.393	12.526	76.648	12.848	24.923	18.697	54.571	18.661	100.064	15.759	9.110	216.187
		11	322.524	12.195	75.804	12.281	26.577	18.612	53.301	17.725	101.921	15.719	8.956	212.578
		12	325.217	11.970	75.397	12.194	25.561	18.680	52.574	17.242	100.709	15.743	9.415	213.737
DGT - F	DGT-F-1	13	158.490	10.705	74.350	7.316	25.110	18.043	54.176	18.623	91.057	9.677	7.071	167.248
		14	162.488	10.085	74.377	7.135	25.198	17.388	52.504	17.265	91.003	9.870	6.826	169.563
		15	156.645	11.445	74.877	7.393	24.864	17.557	54.650	18.300	91.628	9.180	7.284	168.148
	DGT-F-2	16	124.012	11.051	76.423	7.911	25.092	18.198	57.894	18.171	103.041	10.620	8.916	175.250
		17	128.127	11.869	77.577	8.086	26.065	17.538	55.120	19.922	110.009	10.120	8.826	172.173
		18	120.841	10.250	78.059	8.024	24.327	18.198	56.302	17.534	108.546	10.219	9.137	171.313
	DGT-F-3	19	114.004	10.625	79.231	8.505	26.223	18.222	54.309	19.182	104.567	11.939	8.568	187.189
		20	110.310	10.673	79.518	8.562	25.523	17.989	54.820	20.504	102.106	11.001	8.401	186.272
		21	112.847	10.447	79.499	8.512	26.683	17.487	54.375	20.599	99.219	11.162	8.580	189.034
	DGT-F-4	22	166.412	13.199	81.659	9.676	27.157	19.318	66.364	20.737	115.534	12.668	9.025	195.398
		23	160.867	12.718	81.418	9.541	26.972	17.946	67.615	21.340	118.233	11.631	9.232	192.527
		24	163.232	12.362	81.702	9.627	25.848	17.980	66.015	22.173	118.344	12.015	9.180	191.857
SF - T	SF-T-1	25	495.972	13.931	32.964	4.497	17.270	13.992	82.517	24.896	86.401	21.203	5.353	251.367

		26	502.463	14.129	33.480	4.755	17.645	14.586	84.938	24.349	80.265	21.669	5.367	257.343
		27	498.646	12.932	32.572	4.615	17.425	12.620	84.133	26.465	78.180	21.326	5.245	253.230
		28	778.582	12.428	40.367	6.358	21.652	11.855	92.517	29.127	91.360	24.181	6.187	322.804
	SF-T-2	29	774.427	12.209	40.904	6.602	21.951	11.945	94.938	28.132	93.108	24.884	6.466	317.741
		30	779.396	13.150	39.485	6.330	21.962	12.340	94.133	30.496	90.563	24.067	6.369	315.018
		31	798.091	13.859	44.099	6.926	21.847	13.213	85.287	33.010	91.571	25.294	7.086	337.399
	SF-T-3	32	792.729	13.898	42.322	7.114	21.728	12.413	86.263	32.955	89.598	24.575	7.052	328.127
		33	799.519	14.351	43.108	7.025	21.916	13.385	84.898	33.491	95.119	25.334	7.789	330.880
		34	765.108	14.336	41.241	6.453	20.034	12.601	86.950	31.287	95.812	26.802	6.513	352.408
	SF-T-4	35	760.368	14.042	39.913	6.034	20.313	12.698	88.392	31.397	97.597	24.979	6.951	348.037
		36	765.286	14.357	39.569	6.087	20.792	12.934	87.739	32.135	94.606	25.438	6.842	344.366
		37	309.915	12.489	51.706	6.585	26.702	15.372	85.490	24.728	73.958	22.333	5.264	268.891
	SF-F-1	38	303.472	12.546	52.157	6.163	27.184	14.885	82.171	24.080	74.235	21.639	5.491	263.594
		39	302.975	11.428	51.798	6.406	25.898	16.032	80.488	25.162	72.413	20.415	5.317	266.545
		40	326.572	11.916	57.344	16.664	26.825	15.987	72.354	26.672	88.096	26.981	5.856	323.808
	SF-F-2	41	322.241	12.312	56.448	16.750	27.965	17.033	69.970	28.342	86.043	26.801	6.000	317.673
SF - F		42	325.066	11.153	57.659	17.394	27.135	15.697	70.320	27.144	88.822	26.019	6.056	319.646
		43	364.524	14.055	53.736	13.560	31.559	19.497	73.647	25.539	105.431	32.152	8.033	313.016
	SF-F-3	44	362.618	13.774	51.920	13.345	33.295	18.738	74.044	26.953	99.196	32.466	8.324	311.930
		45	369.915	14.526	53.088	13.091	32.152	19.813	72.237	23.333	97.659	32.705	7.813	314.744
		46	421.903	15.431	57.321	13.266	29.169	17.963	62.616	29.609	114.824	28.195	7.447	337.780
	SF-F-4	47	420.209	16.671	57.860	12.637	29.873	17.034	60.478	30.896	112.778	30.202	7.083	338.817
		48	427.569	15.173	57.629	12.814	31.619	18.771	60.904	30.349	113.209	30.327	7.396	344.678
		49	688.061	31.928	174.663	34.525	169.910	89.509	80.101	46.542	67.248	64.553	7.304	322.844
	JS-T-1	50	682.962	32.116	178.275	34.884	172.529	90.029	77.631	49.587	69.526	62.697	7.786	321.014
		51	685.886	32.615	171.820	33.747	170.550	86.072	78.806	44.748	67.937	73.600	7.564	319.880
JS - T		52	904.766	38.063	249.781	61.930	323.403	112.956	177.545	56.273	37.665	77.539	4.407	321.423
	JS-T-2	53	908.137	39.455	243.422	60.335	325.525	105.917	178.676	56.471	39.563	79.504	4.492	324.637
		54	901.697	40.143	245.787	62.934	333.383	107.493	176.372	57.812	36.700	79.053	4.082	318.371
	JS-T-3	55	853.123	14.583	226.818	23.394	236.669	94.422	173.092	75.940	22.000	89.454	3.115	243.039

		56	850.488	15.075	222.463	21.142	240.759	91.083	171.385	75.872	22.830	89.929	3.352	248.527
		57	845.888	15.903	221.467	24.126	238.506	97.968	175.958	75.439	23.123	89.025	3.035	243.846
		58	1003.433	42.923	244.377	42.042	277.131	122.812	143.732	75.331	39.827	90.831	4.121	353.115
	JS-T-4	59	1007.553	45.631	250.633	41.227	269.717	129.948	149.445	81.925	40.977	91.917	3.995	357.329
		60	997.679	43.732	248.007	42.088	273.453	123.488	145.742	79.621	40.482	91.862	4.043	356.758
		61	430.752	31.053	193.766	31.506	116.676	67.593	145.320	44.338	101.443	65.242	8.207	323.858
	JS-F-1	62	433.447	30.190	193.034	30.997	114.451	69.579	141.461	42.883	99.782	64.463	8.237	318.217
		63	432.961	31.065	193.404	32.455	113.724	70.142	145.539	42.613	98.369	64.004	8.832	322.258
		64	453.140	29.602	204.703	24.526	131.201	71.098	103.155	52.312	101.551	66.497	8.835	317.895
	JS-F-2	65	444.500	29.235	208.866	23.790	136.978	73.925	98.912	47.966	96.335	66.021	8.721	319.885
JS - F		66	440.378	31.419	197.478	25.127	129.132	72.502	98.902	51.169	99.223	65.003	9.095	323.623
		67	451.790	30.808	211.280	22.365	135.950	69.766	105.926	46.167	98.980	67.146	8.458	290.009
	JS-F-3	68	445.474	32.865	216.043	20.899	131.047	67.995	98.775	43.233	97.020	66.024	8.671	297.821
		69	448.121	31.617	213.426	21.438	134.243	70.063	101.405	42.442	97.167	66.236	8.568	292.791
		70	491.936	34.047	222.581	22.593	131.430	67.438	113.128	53.713	95.551	72.254	7.412	364.178
	JS-F-4	71	495.657	33.022	224.466	24.224	130.195	67.022	119.186	49.038	94.946	73.058	7.002	361.472
		72	490.444	33.548	222.706	23.175	125.609	73.179	116.123	50.882	96.467	73.546	7.725	365.279
